# Comparative analysis of different approaches for dealing with candidate regions in the context of a genome-wide association study

**DOI:** 10.1186/1753-6561-3-s7-s93

**Published:** 2009-12-15

**Authors:** Francesca Lantieri, Min A Jhun, Jungsun Park, Taesung Park, Marcella Devoto

**Affiliations:** 1 The Children's Hospital of Philadelphia, 3615 Civic Center Boulevard, Philadelphia, Pennsylvania 19104, USA; 2 Dipartimento di Scienze della Salute, sezione di Biostatistica, Universita' degli Studi di Genova, via Pastore 1, 16132 Genoa, Italy; 3 Interdisciplinary Program in Bioinformatics, Seoul National University, Seoul, 56-1 Shillim-Dong, Kwanak-Gu, Seoul, 151-742, Korea; 4 Department of Statistics, Seoul National University, Seoul, 56-1 Shillim-Dong, Kwanak-Gu, 151-747, Korea; 5 Department of Pediatrics and CCEB, University of Pennsylvania, 3615 Civic Center Boulevard, Philadelphia, Pennsylvania 19104, USA; 6 Dipartimento di Medicina Sperimentale, Universita' La Sapienza, Viale Regina Elena 324, 00161, Roma, Italy

## Abstract

Genome-wide association studies (GWAS) test hundreds of thousands of single-nucleotide polymorphisms (SNPs) for association to a trait, treating each marker equally and ignoring prior evidence of association to specific regions. Typically, promising regions are selected for further investigation based on *p*-values obtained from simple tests of association. However, loci that exert only a weak, low-penetrant role on the trait, producing modest evidence of association, are not detectable in the context of a GWAS. Implementing prior knowledge of association in GWAS could increase power, help distinguish between false and true positives, and identify better sets of SNPs for follow-up studies.

Here we performed a GWAS on rheumatoid arthritis (RA) patients and controls (Problem 1, Genetic Analysis Workshop 16). In order to include prior information in the analysis, we applied four methods that distinctively deal with markers in candidate genes in the context of GWAS. SNPs were divided into a random and a candidate subset, then we applied empirical correction by permutation, false-discovery rate, false-positive report probability, and posterior odds of association using different prior probabilities. We repeated the same analyses on two different sets of candidate markers defined on the basis of previously reported association to RA following two different approaches. The four methods showed similar relative behavior when applied to the two sets, with the proportion of candidate SNPs ranked among the top 2,000 varying from 0 to 100%. The use of different prior probabilities changed the stringency of the methods, but not their relative performance.

## Background

Genome-wide association studies (GWAS) to identify the genetic risk factors underlying complex disease are now feasible thanks to advances in genotyping technology and the development of commercial products featuring panels with hundreds of thousands of single-nucleotide polymorphisms (SNPs). A common approach is to use the GWAS design to detect "promising" trait-associated regions that could undergo further investigation. Typically, the top ranked markers are selected for follow-up analysis based on *p*-values obtained from simple tests of association, such as the 1 degree-of-freedom chi-square test on allele frequency difference between cases and controls. For several diseases, some candidate genes may have already been identified by linkage or association studies, or can be suggested on the basis of functional or other biological, rather than statistical, evidence. Such loci may exert only a weak, low-penetrance role on the trait, producing modest evidence of association. In the context of a genome-wide study, the significance of markers in these regions could be low and thus undetectable. GWAS typically ignore any prior knowledge that may support evidence of association to specific regions by treating each marker equally. Incorporating this information into GWAS could increase power, help distinguish between false and true positives, and identify better informed sets of SNPs for follow-up studies.

The *HLA-DRB1 *gene has long been known to be a major rheumatoid arthritis (RA) susceptibility locus [1]. More recently, variants of the *PTPN22 *gene have been reported to be associated to RA [2,3]. Common genetic variants at *TRAF1 *and *C5 *[4] and a haplotype at *STAT4 *have been described in association to RA [5]. Besides these, several other genes have been proposed and tested for association to RA with controversial results.

Different ways to deal with markers in candidate genes in the context of genome-wide studies have been suggested [6-8]. Here we selected specific genomic regions as RA candidate loci using two different approaches and compared the relative performance of some of these methods applied to the RA dataset analyzed by Plenge et al. [4] (Problem 1 of Genetic Analysis Workshop 16 (GAW16)).

## Methods

### Candidate regions

On the basis of previously reported association to RA and following two different approaches, we have defined two sets of RA candidate markers: a more inclusive and exhaustive "gene-based" set, and a more selective "SNP-based" set.

Candidate Subset 1 included 64 genes defined as associated to the broad phenotype of RA in the Genetic Association Database [9,10]. SNPs included in the prioritized subset were those located in a candidate gene and a region of 5 kb on either side of it.

Set 2 was chosen through a review of recently (2006-2008) published studies of RA [2-5]. Markers described in these papers or in their references were included in the Set 2. There were more than 50 SNPs reported as showing association with RA, among which 24 SNPs were genotyped in GAW16. These SNPs and 14 additional ones with pair-wise *r*^2 ^with them greater than 0.5 were selected, leading to a total of 38 candidate SNPs. In addition, we searched RA-related SNPs from NCBI OMIM database [11] and retrieved from the HapMap data those SNPs whose pair-wise *r*^2 ^with them greater than 0.5, for a total of 51 genes. Thus, 89 SNPs were included in the prioritized subset, and the remaining SNPs were defined as the non-prioritized subset.

We excluded from all analyses the HLA region genes on chromosome 6 (from 29,794,096 bp to 33,209,868 bp) because HLA genes, and in particular DR alleles, have already been unequivocally demonstrated to be strongly associated to RA.

The genotypes at 545,080 SNPs from the Illumina 550k chip were available for 868 North American Rheumatoid Arthritis Consortium cases and 1194 controls. SNPs that failed genotyping in more than 10% of all individuals or presenting a minor allele frequency (MAF) lower than 1% were discarded. Missing genotypes at more than 10% of markers was set as a sample exclusion condition, but none of the individuals were removed.

After filtering for low MAF and low genotyping rate, and excluding markers from the HLA region, 515,179 SNPs remained. Of these, 681 SNPs localized in 55 candidate genes in Set 1, and 72 were among the candidate SNPs in Set 2. The complete lists of SNPs and genes included in the two prioritized subsets are available from the authors. The complementary subsets of markers were considered to be random SNPs routinely genotyped in GWAS.

### Statistical methods

We have applied four different methods to prioritize results from candidate regions in the context of a genome-wide study, in addition to standard GWAS.

These methods included a Bayesian approach to calculate posterior odds for association (henceforth referred to as PO), assigning different prior probabilities to the selected candidate marker subset and to the routinely genotyped marker subset [6]. Initially, we specified a prior probability of *p *= 0.1 for the candidate region markers and of *p *= 0.00018 for the other markers (PO1). These values were chosen so that a candidate marker significant at *p *= 0.01 and a routine marker significant at *p *= 0.00001 yield similar values for the PO, as suggested by Curtis et al. [6]. To investigate the effect of different prior probabilities, we also assigned a less confident prior probability of association of *p *= 0.01 to the candidate SNPs subset (PO2).

Next we used the approach based on the false-positive report probability (FPRP) described by Wacholder et al. [7]. The FPRP is the probability of having no true association between a genetic variant and disease given a statistically significant finding. This can be calculated based on the prior probability of real association, the observed *p*-value, and the statistical power of the study, which in turn depends on the sample size and the odds-ratio under the alternative hypothesis. The odds-ratio under the alternative hypothesis was fixed at 1.2 and two different prior probabilities of 0.1 (FPRP1) and 0.01 (FPRP2) for candidate gene markers and 0.00018 for random markers were considered. We used the formula described by Wacholder et al. [7] to calculate the FPRP.

We also utilized a prioritized subset analysis (PSA) and applied the false-discovery rate (FDR) procedure separately to the subset of candidate markers and to the complementary subset of random markers, as suggested by Li et al. [8].

Finally, we calculated empirically corrected *p*-values by permutation for the whole subset of random SNPs and for the candidate SNP subset separately (henceforth referred to as EMP), by swapping the case/control label of individuals (assuming they are interchangeable under the null). A total of 10,000 permutations were ran on each SNP by means of the (max) T procedure implemented in the software PLINK [12,13]. This procedure controls for the number of SNPs tested by comparing each observed test statistic against the maximum of all permuted statistics (i.e., over all SNPs) for each single replicate.

Standard case-control genome-wide association analysis was carried out using the 1-df allelic test from PLINK [13]. To implement the PO1 and PO2 procedures, *p*-values were converted into PO values for association using software provided by Curtis et al. [6,14]. The FPRP was calculated using software provided by Wacholder et al. [7]. To apply the FDR recommended by Li et al. [8], the procedure described by Benjamini and Hochberg [15] and implemented in PLINK was used.

## Results

The genome-wide analysis conducted on the 515,179 SNPs that passed quality control revealed that 66 SNPs were associated to RA with allelic test *p*-values < 10^-7^, which remained significant at α = 0.05 after Bonferroni correction for multiple tests. None of those SNPs was located in any of the 55 selected candidate genes of Set 1, while six were included in the candidate Set 2 (rs10760130, rs1953126, rs2900180, rs3761847, and rs881375 in the region around *TRAF1 *on chromosome 9, and rs2476601 in *PTPN22 *on chromosome 1).

To identify regions worth of further analyses in follow-up studies, we arbitrarily chose the first 2,000 SNPs based on the ranking derived by the standard analysis and the other approaches described in the Methods section. Results thus obtained within each of the two candidate sets are compared.

### Candidate set 1

The 2,000 most significant SNPs following standard genome-wide analysis had allelic test *p*-values that ranged between 3.25 × 10^-4 ^and 1.10 × 10^-15^. Even though not significant at the genome-wide level, two SNPs from this group were located in candidate genes, specifically rs6435203 in *CTL4A *(*p *= 2.56 × 10^-4^) and rs10480340 in *TRB@ *(*p *= 1.70 × 10^-4^).

**Figure 1:**
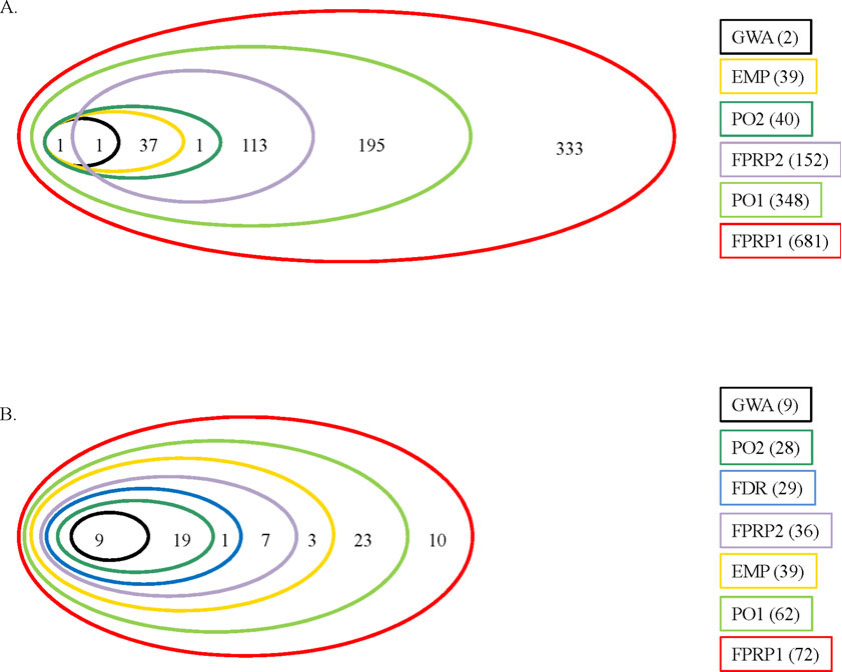
**Top ranked SNPs detected by each method**. Venn diagram with the number of candidate SNPs ranked among the top 2,000 markers by each method in Candidate Sets 1 (A) and 2 (B). GWA, standard allelic test; FDR, PSA followed by FDR [8]; EMP, PSA followed by empirical correction; PO1 and PO2, posterior odds [6] with prior probabilities for candidate SNPs of 0.1 and 0.01; FPRP1 and FPRP2, false-positive report probability [7] with prior probabilities for candidate SNPs of 0.1 and 0.01.

When applying the FDR procedure separately in the candidate SNP subset and in the complementary subset of random markers [8], none of the candidate SNPs ranked among the top 2,000 or had FDR < 0.05. The list of the 2,000 top-ranked SNPs obtained by PSA/FDR was almost identical to that obtained on the basis of their nominal *p*-values. Following separate permutation analysis on the subset of random SNPs and on the candidate SNP subset (EMP), only 575 SNPs showed an empirical *p*-value lower than 1 and could thus be ranked, including 39 SNPs from 22 candidate genes. In the ranking based on the PO values, specifying a prior probability of *p *= 0.1 for candidate gene markers and of *p *= 0.00018 for the other routinely genotyped markers [6], 348 of the 2,000 top ranked SNPs were located in 49 of the candidate genes. Using a prior probability of *p *= 0.01 for the candidate gene markers reduced their number to 40 SNPs from 19 genes. All 681 candidate gene SNPs (with just one with FPRP less than 0.05) were included in the top 2,000 markers using FPRP1, and only 152 using FPRP2 [7] (Figure 1).

In conclusion, 3,318 different SNPs were ranked among the first 2,000 SNPs by at least one method, including all 681 SNPs located on the selected candidate genes. Only 345 SNPs were ranked among the top 2,000 by all methods, and none of these were located on a candidate gene.

### Candidate set 2

Nine candidate SNPs from Set 2 were among the top 2,000 SNPs ranked with the standard genome-wide analysis.

The PSA/FDR method increased the number of candidate SNPs among the top ranked to 29, with the inclusion of 20 additional SNPs, with nominal *p*-values ranging from 3 × 10^-2 ^to 1 × 10^-12^. Of these, 26 had FDR < 0.05, while in the standard analysis only eight candidate SNPs had significant FDR.

Ranking based on *p*-values empirically corrected by permutation for the subsets of non-candidate and candidate SNPs separately raised the number of candidate SNPs among the top ranked SNPs to 39, including 11 with empirical *p*-values lower than 0.05. In this analysis, the total number of SNPs with empirical *p*-values lower than 1 was 500.

The number of top-ranked candidate SNPs further increased to 62 based on the POs ranking under PO1. Under PO2, this number was only 28.

Using FPRP1, all 72 candidate SNPs were included among the top 2,000, although only 14 had FPRP < 0.05. When the prior probability of the candidate SNPs was changed to 0.01 (FPRP2), only 36 of them were retained. In conclusion, the PO method was more stringent than the FPRP method under both sets of prior probabilities (Figure 1).

Overall, 2,798 different SNPs were ranked among the first 2,000 SNPs by at least one method, including all 72 candidate SNPs. Nine candidate SNPs in particular were among the 319 SNPs ranked among the top 2,000 by all methods.

## Discussion

With respect to selection of SNPs for follow-up studies, the standard genome-wide analysis yielded the lowest number of candidate SNPs among the top 2,000 for all the methods applied to Set 2, while the PSA/FDR method was even less inclusive for Set 1. The application of methods designed to prioritize results from selected regions generally increased the number of candidate SNPs that would undergo follow-up analyses. The four methods showed the same relative performance in the two candidate subsets, with the PSA/FDR approach being the least inclusive, followed by the empirical correction performed separately on the candidate subsets and the random subsets, and finally by the POs method using the prior probability *p *= 0.1. In both candidate subsets, the FPRP approach with prior probability set at 0.1 ranked all candidate SNPs among the top 2,000 markers and was thus more permissive than the other methods.

The prior probabilities used for PO1 and FPRP1 (0.1 for candidate SNPs and 0.00018 for random SNPs) result in a 556-fold greater confidence on causality of candidate SNPs compared to random SNPs. To evaluate the impact of these parameters, we also considered a less confident prior for the candidate SNP of *p *= 0.01. Under these conditions, PO2 and FPRP2 were less inclusive, as expected, yielding an even smaller number of candidate SNPs among the top 2,000 than EMP. However, their relative performance remained unchanged.

The different methods in Set 2 provided more homogenous results than in Set 1 (Figure 1). The number of candidate SNPs that were ranked among the top 2,000 by the different methods ranged from 0 to 681 (100%) in Set 1, and from 28 (39%) to 72 (100%) in Set 2. When ranking the top 1,000 SNPs only, those figures ranged from 0% to 63% in Set 1 and from 28% to 97% in Set 2, indicating that the candidate SNPs from Set 2 were more highly ranked on average than those from Set 1. In addition to comprising a smaller number of SNPs, our hypothesis is that this may be due to the fact that Set 2 included stronger candidates following a more careful review of the recent literature, while Set 1 included every gene reported at least once in the literature as associated to RA. Specifically, Set 2 was SNP-based, so that actual SNPs reported as associated with RA were investigated (or SNPs in linkage disequilibrium with them), while Set 1 was gene-based, so that SNPs localized in a candidate gene, but not necessarily related to RA, could have lowered the power to detect association. As a matter of fact, only 10% of SNPs from the Candidate Set 1 had nominally significant *p*-values < 0.05, while 42% of SNPs from Set 2 were nominally significant at the same α.

Bayesian methods are particularly intriguing as a way to deal with multiple tests in GWAS. It has been claimed that in interpreting observed associations the prior credibility of the hypotheses is more relevant than the number of tests performed [16]. The methods by Curtis et al. [6] and Wacholder et al. [7], while inspired by a Bayesian approach, are not fully Bayesian. They do not specify a prior distribution for the effect size, which is assumed as known in the FPRP method (dichotomized as presence or absence of effect) [7]. Curtis et al. [6] instead used the ratio of likelihoods maximized under the alternative and null hypotheses as proxies for the distribution that would have been obtained over the universe of alternative and null hypotheses. In this light, additional methods, such as the Bayesian false-discovery probability recently proposed by Wakefield et al. [17] should be considered and investigated as an interesting alternative approach.

## Conclusion

The results obtained here are dependent on a number of arbitrary choices, such as the set of candidate genes and SNPs selected, the prior probabilities used in the analyses, and the number of SNPs selected for follow-up analyses. Given the present conditions, the relative performance of the methods tested was similar in the two candidate subsets, even though the selection criteria for the two sets were very different. However, in neither of the two candidate subsets do we know which variant is truly disease-associated. Thus we cannot say if the association with candidate markers detected using these methods reflects a real increase in power to detect weak but true association. More detailed analyses of simulated data under different scenarios for variable ranges of the model parameters are needed to better evaluate these methods' relative performances.

## List of abbreviations used

EMP: Empirical *p*-value calculated by permutation; FDR: False-discovery rate; FPRP: False-positive report probability; GAW16: Genetic Analysis Workshop 16; GWAS: Genome-wide association study; MAF: Minor allele frequency; PO: Posterior odds; PSA: Prioritized subset analysis; RA: Rheumatoid arthritis; SNPs: Single-nucleotide polymorphisms

## Competing interests

The authors declare that they have no competing interests.

## Authors' contributions

FL participated in the design of the study, selected the candidate gene Set 1, performed the statistical analysis and drafted the manuscript. MAJ participated in the design of the study, selected the candidate gene Set 2, performed the statistical analysis, and helped to draft the manuscript. JP performed the raw data preprocessing participated in the data analysis and discussion followed. TP conceived of the study, participated in its design and coordination, and helped to draft the manuscript. MD conceived of the study, participated in its design and coordination, and helped to draft the manuscript. All authors read and approved the final manuscript.
